# Succinate Supplement Elicited “Pseudohypoxia” Condition to Promote Proliferation, Migration, and Osteogenesis of Periodontal Ligament Cells

**DOI:** 10.1155/2020/2016809

**Published:** 2020-03-10

**Authors:** Huimin Mao, Andi Yang, Yunhe Zhao, Lang Lei, Houxuan Li

**Affiliations:** ^1^Nanjing Stomatological Hospital, Medical School of Nanjing University, Nanjing, China 210008; ^2^Central Laboratory of Stomatology, Nanjing Stomatological Hospital, Medical School of Nanjing University, Nanjing, China 210008

## Abstract

Most mesenchymal stem cells reside in a niche of low oxygen tension. Iron-chelating agents such as CoCl_2_ and deferoxamine have been utilized to mimic hypoxia and promote cell growth. The purpose of the present study was to explore whether a supplement of succinate, a natural metabolite of the tricarboxylic acid (TCA) cycle, can mimic hypoxia condition to promote human periodontal ligament cells (hPDLCs). Culturing hPDLCs in hypoxia condition promoted cell proliferation, migration, and osteogenic differentiation; moreover, hypoxia shifted cell metabolism from oxidative phosphorylation to glycolysis with accumulation of succinate in the cytosol and its release into culture supernatants. The succinate supplement enhanced hPDLC proliferation, migration, and osteogenesis with decreased succinate dehydrogenase (SDH) expression and activity, as well as increased hexokinase 2 (HK2) and 6-phosphofructo-2-kinase/fructose-2,6-biphosphatase 3 (PFKFB3), suggesting metabolic reprogramming from oxidative phosphorylation to glycolysis in a normal oxygen condition. The succinate supplement in cell cultures promoted intracellular succinate accumulation while stabilizing hypoxia inducible factor-1*α* (HIF-1*α*), leading to a state of pseudohypoxia. Moreover, we demonstrate that hypoxia-induced proliferation was G-protein-coupled receptor 91- (GPR91-) dependent, while exogenous succinate-elicited proliferation involved the GPR91-dependent and GPR91-independent pathway. In conclusion, the succinate supplement altered cell metabolism in hPDLCs, induced a pseudohypoxia condition, and enhanced proliferation, migration, and osteogenesis of mesenchymal stem cells in vitro.

## 1. Introduction

Periodontitis is an inflammatory disease notable for loss of periodontal ligament stem cells and supporting tissues [1]. Despite the long-term effort to reconstruct periodontal tissues by guided tissue regeneration, guided bone regeneration, and application of enamel matrix derivatives, periodontal regeneration is still challenging for periodontists [2]. Stem cell-based regenerative approaches, especially application of human periodontal ligament cells (hPDLCs), have attracted much clinical interest [3]. Recently, in a clinical study, transplantation of autologous hPDLCs with bovine-derived bone minerals improved prognosis of periodontal intrabony defects [4]. Such stem cell-based regeneration largely depends on in vitro expansion of stem cells, and this is especially critical in the case of aged patients with periodontitis [5].

Normally, nonproliferated and undifferentiated stem cells grow in a low-oxygen niche (less than 21% oxygen). Oxygen tension in the bone marrow is 1–6% [6], while the normal oxygen tension in the periodontal membrane ranges from 2.9% to 5.7% [7]. Oxygen saturation level is one major factor determining biological activities of stem cells. Hypoxia promotes cell growth and migration, and it helps maintain cell stemness [8, 9]. The effects of hypoxia are primarily mediated by hypoxia-inducible factors (HIFs) [10], which are DNA-binding transcriptional factors and bind to hypoxia-regulated elements (HREs) in the promoter and enhancer of target genes [10]. In normal oxygen condition, HIF-1*α* nuclear translocation is consistently diminished by prolyl hydroxylase 2- (PHD2-) mediated hydroxylation [11].

A hypoxia state can be achieved and maintained by utilizing hypoxic chambers; however, changes of gas media (95% N_2_ and 5% CO_2_) for long-term cell culture are cost- and time-consuming. Therefore, hypoxia-mimicking agents such as cobalt chloride (CoCl_2_) and deferoxamine (DFO) have been tried in the expansion of stem cells [12]; however, the cytotoxicity of this agent is still an issue [8]. In a hypoxic state, cell metabolism shifts from oxidative phosphorylation (OxPhos) to glycolysis for generation of adenosine triphosphate (ATP) and metabolic intermediates [13]. Accumulation of citrate and succinate, intermediates of the tricarboxylic acid (TCA) cycle, can be commonly observed in cells cultured in hypoxia, as the biological activity of key speed limit enzymes, isocitrate dehydrogenase (IDH) and succinate dehydrogenase (SDH), can be dampened by a hypoxia state and HIF-1*α* stabilization [14].

Succinate produced in the mitochondrial matrix can be exported to the cytosol via the dicarboxylate carrier SLC25A10 [15], and high concentration of succinate in the cytosol stabilizes HIF-1*α*, thereby promoting HIF-1*α*-dependent genes even O_2_ concentration was high in the cytosol [16]. Moreover, succinate in the cytoplasm can be released into the extracellular space and act as a metabolic signal. Succinate may bind to G-protein-coupled receptor 91 (GPR91), which is also named as succinate receptor 1 (SUCNR1), and trigger a cascade of biological events [13]. In macrophages, succinate activation may polarize macrophages into a proinflammatory subtype (M1 macrophages) [16]. In addition, succinate may enhance migration of stem cells after activating succinate receptors [17]. However, whether the succinate can be utilized to mimic a pseudohypoxia state in the cytosol and promote proliferation of stem cells has never been reported. In our present study, we discovered for the first time that levels of succinate were elevated in hPDLCs cultured under hypoxia and exogenous succinate can be utilized to mimic the hypoxia state and reprogram cell metabolism in hPDLCs.

## 2. Materials and Methods

### 2.1. Isolation and Identification of hPDLCs

hPDLCs were separated and cultured using explant techniques. Briefly, teeth were obtained from premolars of subjects (mean age: 13 years old) needing orthodontic treatment, and informed consent was acquired from each participant. The PDL tissues were scalped from the middle-third of the root surface and cultured in Dulbecco's modified Eagle's medium (DMEM, Gibco, USA) containing 10% *v*/*v* fetal bovine serum (FBS, Gibco, USA) and 1% penicillin/streptomycin at 37°C in a humidified atmosphere containing 5% CO_2_. The medium was changed after 3 days, and the outgrown cells were passaged at approximately 80% confluence. Cells at the 3rd to 5th passages were used for the study.

### 2.2. Flow Cytometry

hPDLCs were identified by flow cytometry using antibodies against CD11b, CD90, CD45, and CD29. hPDLCs (2.5 × 10^5^/mL, 3rd passage) were placed in the 1.5 mL Eppendorf tubes and washed with PBS twice. Next, fluorescein isothiocyanate- (FITC-) conjugated or phycoerythrin- (PE-) conjugated anti-CD11b, anti-CD90, anti-CD45, and anti-CD29 antibodies were added to hPDLC samples and incubated at room temperature in the dark for 30 min. The percentages of cells positively stained with CD11b, CD90, CD45, and CD29 were assessed with fluorescence-activated cell sorting.

### 2.3. Hypoxia/Succinate Treatment

1 × 10^6^ cells were plated in 10 cm. Cells were cultured in either the normal oxygen condition or the hypoxia condition (1% oxygen) in the hypoxic chamber (Thermo Scientific, USA). If oxygen tension rose above the desired level, nitrogen gas was automatically injected into the system to replace the excess oxygen. For the succinate supplement group, sodium succinate dibasic hexahydrate (Sigma-Aldrich, USA) was added to the culture medium at the designated concentration (1, 5, or 25 mM). To inhibit HIF-1*α* activity, hPDLCs were pretreated with the HIF-1*α*-specific inhibitor BAY 87-2243 (5 nM; Selleck, USA) for 3 h.

### 2.4. Cell Proliferation Assay

hPDLCs were seeded at a density of 2 × 10^3^cells/well into 96-well plates and cultured for 24 h. To determine the effects of succinate or hypoxia on the proliferation of hPDLCs, cells were treated with succinate (0, 1, 5, and 25 mM) for 24 h, 48 h, 72 h, and 96 h or hypoxia (1% O_2_) for 24 h. Cell proliferation was assessed with Cell Counting Kit-8 (CCK-8) (Dojindo, Japan). In CCK-8 analysis, CCK-8 was added for 2 h at 37°C and the absorbance was determined using a microplate reader with 450 nm wavelengths.

### 2.5. Wound-Healing Migration Assay

hPDLCs were plated and grown at 3 × 10^5^ cells/well in 6-well plates prepared with five straight lines on the back for 24 h. Then, scratching was mechanically executed with a 20 *μ*L pipetting spear perpendicular to the five base lines. Cells were incubated for another 24 h with succinate (0, 1, 5, and 25 mM) or hypoxia (1% O_2_) treatment and visualized with an inverted microscope (Nikon, Japan).

### 2.6. Alkaline Phosphatase (ALP) and Alizarin Red S Staining

hPDLCs were seeded in 24-well plates at a density of 2.5 × 10^4^ cells/well and incubated in the osteogenic medium, which was supplemented with 50 mg/mL ascorbic acid, 100 nM dexamethasone, and 10 mM *β*-glycerophosphate for 7 days. The medium was changed every 48 h. Cells were washed with phosphate-buffered solution (PBS, Gibco, USA) 3 times and treated with a 4% paraformaldehyde fixative for 30 min. Then, the plates were washed again with PBS and incubated at room temperature using a BCIP/NBT alkaline phosphatase color development kit (Beyotime, China) with corresponding proportions of solution for 30 min according to the manufacturer's instructions.

Calcium deposition was detected by Alizarin Red S staining. hPDLCs were cultured at 5 × 10^4^ cells/well in 12-well culture plates for 21 days in the osteogenic medium. Cells were rinsed twice with PBS and fixed in 10% formaldehyde for 10 min. The cells were then washed and stained with Alizarin Red S (Sigma-Aldrich, USA) for 20 min at room temperature. Excess dye was removed by gently rinsing with deionized water until the background became clear.

### 2.7. Measurement of Succinate Concentration and Succinate Dehydrogenase Activity

The extracellular and intracellular succinate concentrations were measured with a succinate colorimetric assay kit (Megazyme, Ireland) according to the manufacturer's instructions. Briefly, the conditional medium was collected and treated with indicated agents. For measurement of intracellular succinate and succinate dehydrogenase activity, cells were disposed with freezing and thawing cycles to collect cell lysates. Then, cell lysis solution was centrifuged at 1,500 rounds per minute for 2 min to remove debris. Succinate dehydrogenase activity was detected with a succinate dehydrogenase ELISA kit (JinYibai, China) according to the manufacturer's protocol.

### 2.8. Reverse Transcription Polymerase Chain Reaction and Real-Time PCR

hPDLCs were seeded in 6-well plates at a density of 3 × 10^5^ cells/well and treated with hypoxia condition (1% O_2_) or succinate (0, 1, and 5 mmol/L) for 4 h. The total RNA was extracted with the RNeasy Plus Mini Kit (Tiangen, China). Reverse transcription reaction was performed with 1 *μ*g of RNA using a cDNA synthesis kit (Vazyme, China). Then, the cDNA was mixed with the primers (GenScript, China) and Maxima® SYBR Green/ROX qPCR Master Mix (Thermo Fisher Scientific) according to the manufacturer's protocol and subjected to real-time PCR on the ViiA™ 7 Real-Time PCR System (Thermo Fisher Scientific, USA). *β*-Actin was used as the internal reference gene, and data were analyzed with the 2^−ΔΔCt^ method. Results were shown as the relative expression ratio of the target gene to the reference gene. The sequence of primers is listed in Table 1.

### 2.9. Western Blot Analysis

Following the indicated treatments, the collected cells were lysed with RIPA lysis buffer for 15 min on ice. Protein concentration was determined by the Bradford method. Next, protein samples were added with loading buffer (GenScript, China) and heated at 95°C for 10 min after which equal amounts of protein were isolated by 10% sodium dodecyl sulfate polyacrylamide gel electrophoresis (SDS-PAGE) and were transferred to polyvinylidene fluoride membranes. Membranes were blocked with 5% BSA in TBST buffer (150 mM NaCl, 50 mM Tris-HCl, and 0.5% Tween 20 (pH 7.6)) at room temperature for 2 h and incubated with primary antibodies at 4°C for 16 h and secondary antibodies (Thermo Fisher Scientific, USA) at room temperature for 2 h. Finally, membranes were exposed to an ECL reagent (NCM Biotech, China) for detecting signals. Images were shot using a Tanon 6200 Luminescent Imaging Workstation (Tanon, China).

### 2.10. Lentivirus Vector-Mediated GPR91 Knockdown in hPDLCs

To knock down the expression of GPR91 in hPDLCs, cells were transfected for 24 h with GPR91 shRNA (25 nM; GenePharma, China) or scramble shRNA (25 nM; GenePharma, China) as a negative control with Envirus™ (Engreen Biosystem) according to the manufacturer's protocol. The culture medium was replaced with complete culture medium 24 h later. At least 72 h after transfection, cells were used for subsequent testing. The sequences of the shRNAs used in this study were as follows: control shRNA (shNC), 5′-GGAGTTATGCCAATGGAAACT-3′, and GPR91 gene shRNA, 5′-GGAGTTATGCCAATGGAAACT-3′.

### 2.11. Statistical Analysis

All data above are expressed as the means ± standard deviation. All statistical analyses were performed using the SPSS 18.0 statistical software. An unpaired *t*-test or one-way ANOVA was used to evaluate statistical significance of the differences between two groups; SNK post hoc tests were conducted once significant difference was detected among groups. *p* < 0.05 was set as the statistical significance level. All of the statistic graphs were produced with GraphPad Prism 7 (GraphPad Software Inc., USA).

## 3. Results

### 3.1. Hypoxia Promoted hPDLC Proliferation, Migration, and Osteogenesis

**Figure 1:**
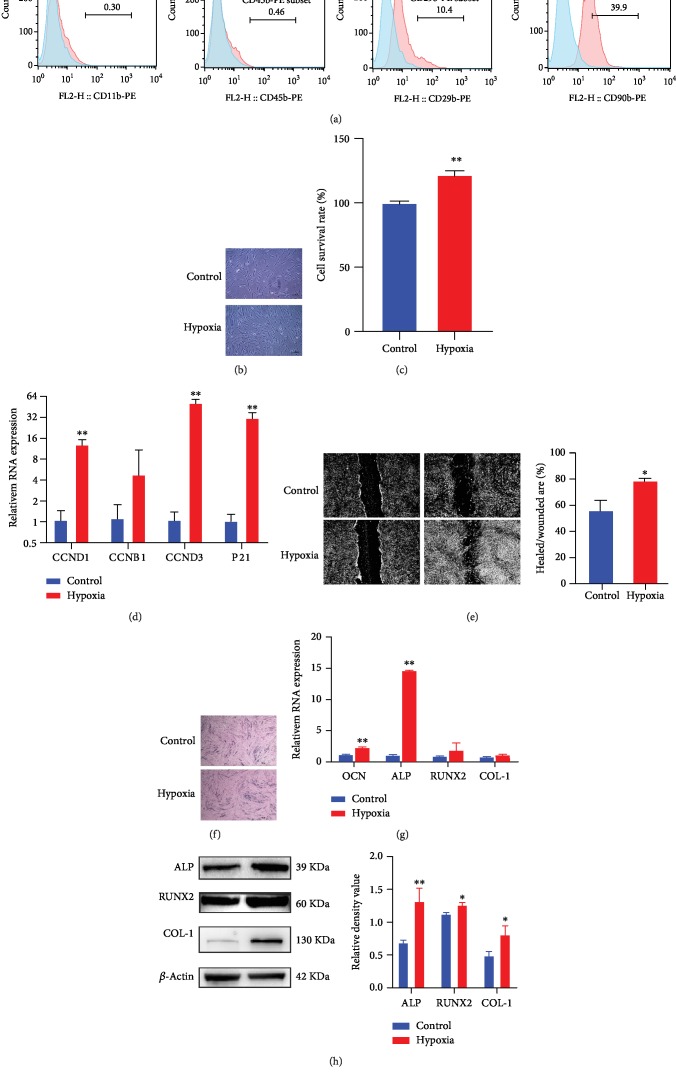
Hypoxia promoted proliferation, migration, and osteogenic differentiation in hPDLCs. Cells separated from periodontal ligaments were characterized by flow cytometry, showing positive expression of markers of MSCs (a). hPDLCs cultured in the normoxia and hypoxia were visualized using a microscope at 24 h (b). Proliferation of hPDLCs was evaluated by the CCK8 assay at 24 h (c). Transcription of cell cycle-related genes was determined by qPCR at 4 h (d). Scratch-healing model was utilized to determine hPDLC migration capacity at 24 h, and the healed/wounded area ratio was calculated (*n* = 3) (e). ALP staining was conducted on cells cultured in the osteogenic medium after 7 days (f). The mRNA of osteogenesis-related genes at 24 h was analyzed by qPCR (*n* = 3) (g). Protein expression of ALP, RUNX2, and Col-1 at 72 h was assayed by western blot; the blots were representative of three independent experiments (h). ^∗^*p* < 0.05, relative to control; ^∗∗^*p* < 0.01, relative to control.

Firstly, expression of surface markers on cells separated from periodontal ligaments was characterized by flow cytometry, showing that cells from the periodontal ligament did not express haematopoietic surface markers (CD11b and CD45), while less than 40% cells exhibited mesenchymal stem cell (MSC) surface markers (CD90 and CD29) (Figure 1(a)); therefore, we characterized the separated cells as human periodontal ligament cells (hPDLCs).

To examine the hypothesis that exogenous succinate can promote biological activities of hPDLCs, we first explored the effects of hypoxic conditioning on hPDLC proliferation, migration, and osteogenic differentiation. When hPDLCs were cultured in a hypoxic state of 1% O_2_ for 24 h, their proliferation was remarkably enhanced under a microscope (Figure 1(b)) and was also confirmed in the CCK-8 analysis (Figure 1(c)). In addition, mRNA transcription of CCND1, CCNB1, CCND3, and P21, molecules that are related to the cell cycle, all increased consistently (Figure 1(d)). Then, we measured hPDLC migration capability by introducing the wound-healing migration test. By measuring the ratio of the healed/wounded area, we observed that the value was significantly increased in the hypoxia group when compared to the normoxia group, indicating that hypoxia promoted cell migration capability (Figure 1(e)). Finally, to determine the effects of hypoxia on the osteogenesis capacity of hPDLCs, the alkaline phosphatase (ALP) stain assay was used. And we found that more ALP-positive cells were observed after culturing cells in hypoxia for 7 days (Figure 1(f)). The effect of hypoxia on hPDLC osteogenic differentiation was further assessed using qPCR, showing that hypoxia treatment enhanced the transcription of osteocalcin (OCN) and ALP, two essential markers for osteoblast differentiation (Figure 1(g)); moreover, culturing hPDLCs in the osteogenic medium in hypoxia enhanced the protein expression of ALP, RUNX2, and collage-1 (COL-1) (Figure 1(h)).

### 3.2. Hypoxia Enhanced Succinate Accumulation and Stabilized HIF-1*α*

**Figure 2:**
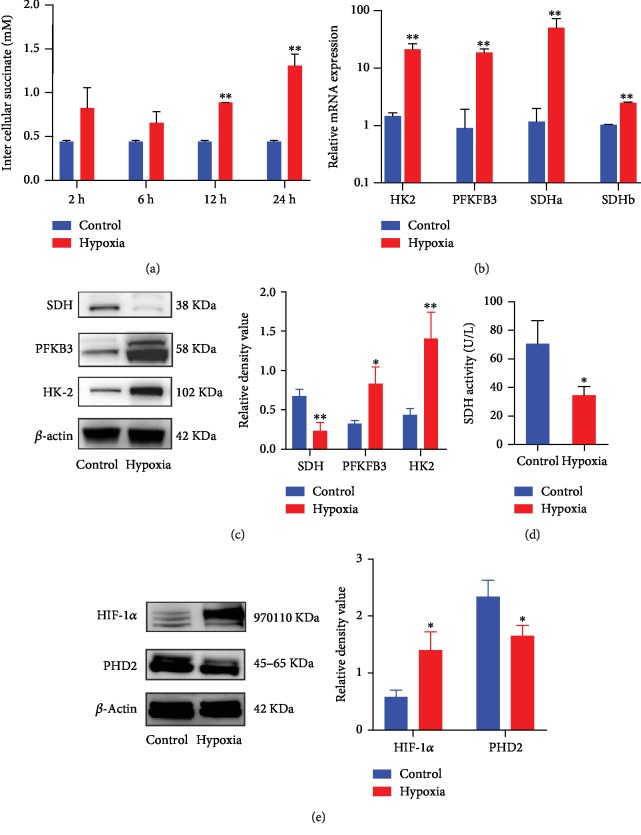
Hypoxia culture enhanced intracellular succinate accumulation and stabilized HIF-1*α*. Levels of intracellular succinate in cell lysates were detected by the succinate colorimetric assay (a). Hexokinase 2 (HK2), 6-phosphofructo-2-kinase/fructose-2,6-biphosphatase 3 (PFKFB3), and succinate dehydrogenase (SDH) transcription at 4 h were determined by qPCR (*n* = 3) (b), while protein expression was detected by western blot at 24 h (c). SDH activity at 6 h was measured by ELISA (d). Protein levels of the PHD2 and HIF-1*α* pathway were measured by western blot at 2 h (e). Blots were representative of 3 independent tests. ^∗^*p* < 0.05, relative to control; ^∗∗^*p* < 0.01, relative to control.

During hypoxia, HIF-1*α* is responsible for the induction of a variety of genes including glycolysis and osteogenesis [18]. Firstly, a colorimetric assay was utilized to examine levels of intracellular succinate, we observed elevation in succinate as early as 2 h, and succinate accumulation in the cytosol of hypoxia-treated cells can be consistently found at all observation time points (Figure 2(a)). In addition, genes involved in the OxPhos and glycolysis were altered by hypoxia with increased transcription of hexokinase 2 (HK2), 6-phosphofructo-2-kinase/fructose-2,6-biphosphatase 3 (PFKB3), and succinate dehydrogenase (SDH) (Figure 2(b)). Moreover, protein levels of HK2 and PFKB3 were both elevated while SDH was diminished (Figure 2(c)). Furthermore, we also detected changes of SDH activity following hypoxia treatment, and we observed that SDH activity was dampened under hypoxia (Figure 2(d)). Oxidation of succinate into fumarate is metabolized by SDH, and impaired SDH activity may lead to succinate accumulation [13]. We also examined alteration of PHD2 and HIF-1*α* and found that levels of PHD2 were decreased while HIF-1*α* was elevated in hypoxia-treated cells (Figure 2(e)).

### 3.3. Succinate Supplement Elicited Intracellular Pseudohypoxia Condition to Promote Proliferation of hPDLCs

**Figure 3:**
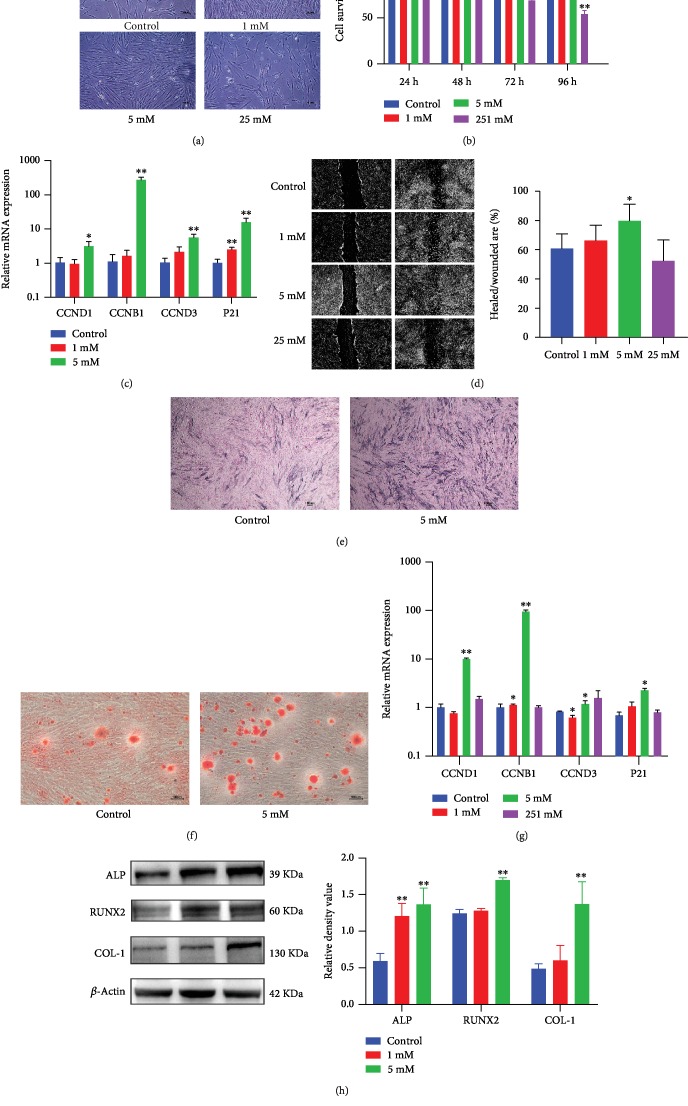
Exogenous succinate promoted proliferation, migration, and osteogenesis in hPDLCs. hPDLCs were treated with 0, 1, 5, or 25 mM of succinate. Cell proliferation was observed under the phase contrast microscope at 24 h (a). The CCK-8 assay was used to quantify cell growth (b). Transcription of cell cycle-related genes at 4 h was detected by qPCR (c). Scratch-healing model was utilized to analyze cell migration at 24 h (d). Osteogenic differentiation was assessed using ALP staining after 7 days and Alizarin Red S Staining after 21 days (e, f). Levels of osteogenesis-related genes in hPDLCs were analyzed by qPCR at 24 h (*n* = 3) (g), and protein expression was detected by western blot at 72 h (h). Blots were representative of 3 independent tests. ^∗^*p* < 0.05, relative to control; ^∗∗^*p* < 0.01, relative to control.

High levels of succinate in the cytosol can reduce PHD activity by product inhibition and promote HIF-1*α* stabilization [19]. We then explored whether the succinate supplement may promote cell proliferation and induce PHD2 degradation and HIF-1*α* stabilization, which has been defined as “pseudohypoxia” [14]. hPDLCs were treated with 0, 1, 5, and 25 mM of succinate for 24 h, 48 h, 72 h, and 96 h, respectively. 1 and 5 mM of succinate increased stem cell proliferation in a microscope (Figure 3(a)) and CCK-8 analysis, while 25 mM reduced proliferation (Figure 3(b)). Similarly, cell cycle and proliferation genes, CCND1, CCNB1, CCND3, and P21, were upregulated after 4 h of succinate treatment (Figure 3(c)). Furthermore, 5 mM succinate increased stem cell migration in the wound scratch model, while 25 mM did not alter the wound-healing process (Figure 3(d)).

Additionally, hPDLCs were cultured in succinate-supplemented (0, 1, 5, and 25 mM) and osteogenic medium for 7 days, and ALP and Alizarin Red Staining were utilized to assess the osteogenic capability of the succinate supplement. ALP-positive cells and calcium deposits were increased in the 5 mM succinate group, suggesting that succinate can promote hPDLC osteogenic differentiation (Figures 3(e) and 3(f)). Moreover, treatment with 5 mM succinate for 24 h boosted mRNA levels of OCN, ALP, RUNX2, and COL-1 (Figure 3(g)), and the succinate supplement promoted the protein expression of ALP, RUNX2, and COL-1 (Figure 3(h)).

**Figure 4:**
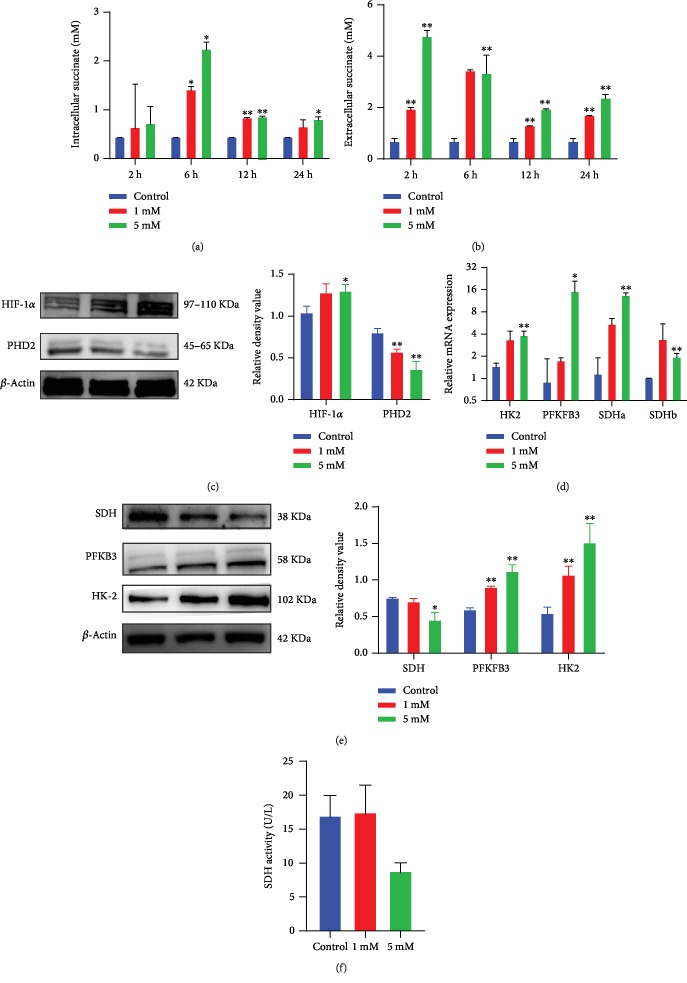
Succinate supplement elicited intracellular succinate accumulation, pseudohypoxia condition, and glycolysis. The intracellular succinate in cell lysates and extracellular succinate in supernatants were assessed (*n* = 3) (a, b). Changes in PHD2 and HIF-1*α* at 2 h after succinate treatment were measured by western blot (c). The mRNA and protein of genes involved in the OxPhos and glycolysis were analyzed at 4 h by qPCR and at 24 h by western blot, respectively (d, e). SDH activity was measured by ELISA after 6 h stimulation (*n* = 3) (f). Blots were representative of 3 independent tests. ^∗^*p* < 0.05, relative to control; ^∗∗^*p* < 0.01 relative to control.

As succinate can be released into the extracellular space from the cytoplasm, succinate may also enter into the cytosol. We then determined levels of intracellular and extracellular succinate after treating hPDLCs with succinate. Succinate concentration in the cytosol climbed in an early phase of succinate stimulation, peaked at 6 h, and then declined later on (Figure 4(a)). In contrast, succinate concentration in the extracellular space decreased over time in succinate-treated cells (Figure 4(b)). In line with elevated succinate in the cytosol, the protein level of HIF-1*α* was increased, whereas the level of PHD was reduced (Figure 4(c)). Then, changes in glycolysis-related genes were analyzed. Both HK2 and PFKFB3 were elevated with the succinate supplement (Figures 4(d) and 4(e)). Furthermore, it was observed that SDH transcription elevated at 4 h while the SDH protein level diminished in succinate-supplemented hPDLCs (Figures 4(d) and 4(e)). Additionally, reduced SDH enzyme activity was found in 5 mM succinate-treated cells (Figure 4(f)). Therefore, our present data suggest that exogenous succinate can be transferred into the cytosol, where it induced PHD suppression, leading to a pseudohypoxia condition and HIF-1*α* stabilization. Therefore, such HIF-1*α* pathway activation was crucial in succinate supplement-induced stem cell proliferation.

### 3.4. HIF-1*α* Activation Was Responsible for the Proliferation of hPDLCs during Hypoxia and Succinate Supplementation

**Figure 5:**
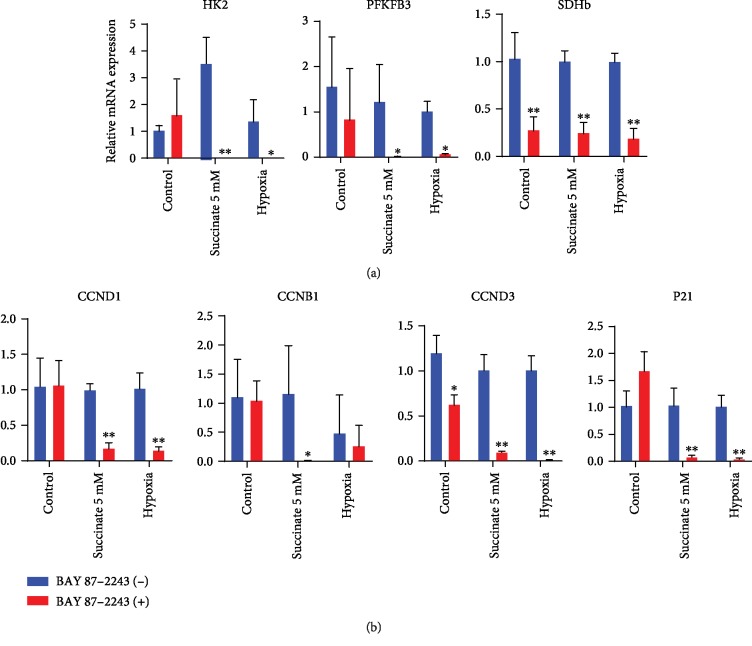
Blocking HIF-1*α* affected genes that were involved in glycolysis and proliferation of hPDLCs during hypoxia incubation and succinate supplementation. hPDLCs were preconditioned with BAY 87-2243 to block HIF-1*α* for 3 h. Changes in metabolic and cell cycle-related genes at 4 h after hypoxia/succinate treatment were measured by qPCR (*n* = 4) (a, b). ^∗^*p* < 0.05, relative to control; ^∗∗^*p* < 0.01, relative to control.

To further characterize whether HIF-1*α* activation triggers proliferation of hPDLCs, we utilized BAY 87-2243, an inhibitor of HIF-1*α*, to inhibit HIF-1*α* pathway activation. After 3 h of BAY 87-2243 pretreatment, both hypoxia and succinate stimulation failed to induce the expression of HK2, PFKFB3, and SDH (Figure 5(a)). Consistently, hypoxia and succinate treatment were incapable of inducing the expression of cell cycle-related genes in the absence of HIF-1*α* (Figure 5(b)). Taken together, our results revealed that both hypoxia and succinate-induced HIF-1*α* stabilization increased glycolysis and proliferation of hPDLCs.

### 3.5. Succinate Acted as an Extracellular Mediator Signal through the GPR91-Dependent and GPR91-Independent Pathway

**Figure 6:**
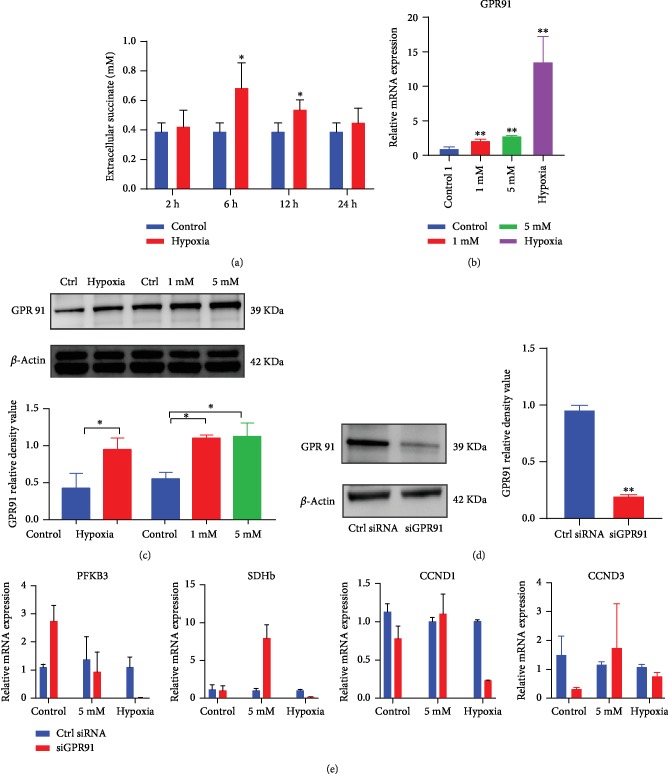
Succinate supplement triggered the GPR91-dependent and GPR91-independent pathway. The extracellular succinate concentration in supernatant was detected in hypoxia-cultured cells (a). The expression of GPR91 was analyzed by qPCR and western blot at 4 h and 24 h, respectively (b, c). Lentivirus-based gene interference was utilized to knock down GPR91 (d). Gene transcription was determined by qPCR at 4 h (e). ^∗^*p* < 0.05, relative to control; ^∗∗^*p* < 0.01, relative to control.

As shown in our study, succinate can move across the cell membrane, i.e., in and out of the cytosol. In addition, succinate can bind to succinate receptors, which are also called GPR91, functioning as an autocrine and paracrine sensor to mediate cell signaling transduction [20, 21]. Before confirming the role of GPR91 in hypoxia, we explored the extracellular succinate concentration under hypoxia. Consequently, we found that succinate concentration in the extracellular milieu in hypoxia-treated hPDLCs was higher at 6 h and the value was not different from that of normoxia control after 24 h (Figure 6(a)). GPR91 was upregulated after 4 h of hypoxia/succinate (5 mM) treatment (Figures 6(b) and 6(c)). These results demonstrated that both endogenous succinate and exogenous succinate can activate GPR91.

To explore the role of GPR91 in hypoxia/succinate-induced stem cell proliferation, we utilized lentivirus-mediated knockdown of GPR91. hPDLCs were transfected with GPR91 siRNA or scramble siRNA (Figure 6(d)). Expression of both metabolism-related genes and cell cycle-related genes in the hypoxia treatment group with GPR91 knockdown was considerably reduced (Figure 6(e)), suggesting that hypoxia-induced metabolism changes and proliferation enhancement was related to the activation of GPR91 through the autocrine pathway of succinate. However, succinate supplement-induced alterations in glycolysis-related and cell cycle-involved genes were not affected by GPR91 knockdown, suggesting that high-level exogenous succinate can bypass GPR91 and directly cross the cell membrane to induce biological effect in a GPR91-independent pathway (Figure 6(e)).

## 4. Discussion

Preserving self-renewal capacity and differentiation capability is essential for the expansion and successful use of stem cells in clinical practices [22]. In this study, we found that hypoxia-promoted hPDLC proliferation and migration were mediated by succinate accumulation and HIF-1*α* pathway activation. Furthermore, for the first time, we discovered that the succinate supplement enhanced proliferation by creating a pseudohypoxia state with PHD2 inhibition and HIF-1*α* stabilization, and we found that hypoxia-induced proliferation enhancement was GPR91-dependent while succinate-elicited proliferation was GPR91-independent.

Similar to previous reports [17], metabolic response to succinate depends on its concentration. High concentration of the succinate supplement still diminished cell viability, and such inhibitory effect may be correlated with the following mechanisms: accumulation of succinate in the cytosol and mitochondria may activate the caspase 3 or caspase 7 apoptotic pathway [23]; succinate can also inhibit 2-oxoglutarate-dependent histone and DNA demethylase enzymes, resulting in epigenetic silencing [24]; furthermore, protein succination, a novel stable posttranslational modification pathway, may also alter protein dynamics [25]; moreover, profuse generation of ROS by intracellular succinate accumulation in the mitochondria may result in macromolecular damage, including DNA, protein, and organelle damage [26].

PHD2 is a nonheme iron-containing molecule, and its activity relies on *α*-ketoglutarate/2-oxoglutarate. PHD2 consistently hydroxylates HIF-1*α* at proline residues 402 and 564 in the cytosol, thereby inhibiting its nuclear translocation. Hydroxylated HIF-1*α* links to the von Hippel-Lindau protein (pVHL), an E3 ubiquitin ligase, leading to degradation of HIF-1*α* in the proteasome. Chelation of Fe^2+^ in this protein by CoCl_2_ and deferoxamine negates normal activity of PHD2; however, since iron is essential for numerous iron-containing proteins, chelating Fe^2+^ may have multiple side effects [27]. PHD2-mediated hydroxylation reaction utilizes dioxygen; one atom is transferred to the proline residue, and the other one is utilized to oxidize *α*-ketoglutarate into succinate with the release of CO_2_ [28, 29]. Both *α*-ketoglutarate and succinate are TCA cycle intermediates [19]. In our present study, succinate supplemented in the cytosol may cross the cell membrane, leading to elevated levels of succinate in the cytoplasm; succinate accumulation reduced PHD2 activity and *α*-ketoglutarate oxidation by product inhibition [30]. Based on the inherent relation between hypoxia-PHD-HIF-1*α* and hypoxic metabolism alterations, we for the first time suggest that the succinate supplement can be utilized to elicit HIF-1*α* pathway activation.

To further unveil the role of succinate accumulation in the cytosol, we observed that inhibiting HIF-1*α* by BAY affected the cell cycle- and glycolysis-related genes under hypoxia and succinate supplementation, indicating that HIF-1*α* mediates hypoxia and succinate-accelerated proliferation in hPDLCs. However, the TCA cycle metabolism was different between hypoxia and succinate supplementation. In the TCA cycle, succinate is converted to fumarate by SDH [19], which utilizes FAD and NAD^+^ as cofactors [31]. Therefore, succinate accumulation may enhance SDH expression and activity by substrate stimulation. In contrast, hypoxia itself dampens SDH activity leading to succinate accumulation [13]. Our data indicates that succinate acts as an intracellular messenger, inducing alterations in gene expression in hPDLCs via inhibiting PHD2 activity to stabilization of HIF-1*α*, creating a state of pseudohypoxia.

Behaviors of MSCs are critically affected by nutrient availability, culturing niche, and metabolic status [26]. Anaerobic glycolysis in the hypoxia condition helps maintain stemness of MSCs while OxPhos may increase osteogenic differentiation [32]. Dampened SDH activity and enhanced HK2 as well as PFKB3 protein expression indicated that the succinate supplement shifted metabolism from OxPhos to glycolysis. Such metabolic changes may help maintain stemness of PDLCs in vitro. Moreover, stemness is metabolically modulated by nutrient-sensing pathways, such as mammalian target of rapamycin (mTOR), adenosine 5′-monophosphate- (AMP-) activated protein kinase (AMPK), and sirtuins (SIRT) [33]. Naturally, MSCs reside in a niche of hypoxia and lack of adequate blood supply. Our current succinate supplement alters normal nutrient balance and availability, since succinate may be metabolized into fumarate to generate adenosine triphosphate and diverted to the “GABA shunt” to fuel the glutamine pathway [34]. Such metabolic changes need to be further confirmed by seahorse-based bioenergetic assay and mass spectrometry-based metabolomics. Further studies are needed to clarify whether the succinate supplement may improve or compromise stemness of PDLFs in vitro.

In our present study, the succinate supplement enhanced proliferation in normal culture and promoted osteogenesis in an osteogenic medium, which was similar to hPDLCs under a hypoxic state [35]. Although a large part of the literature on MSCs demonstrates that hypoxic milieu is in favor of cell expansion and stemness, the effect on osteogenic differentiation is still debated, with both reduced [32, 36] and enhanced [37–39] osteogenic differentiation being reported. Such controversies regarding this important issue may be related to differed culture systems, such as donor species, cell types (embryonic, hematopoietic, and tissue specific stem cells), hypoxia severity, and methods of induction [32, 40]. In our present study, the supplemented succinate may be oxidized into fumarate to produce ATP or diverted to the “GABA shunt” to fuel the glutamine pathway [34], thereby maintaining active glycolysis needed for high rates of proliferation in a pseudohypoxic state at early stages of osteogenesis and enhancing bone matrix protein biosynthesis at later stages. Since metabolic state, i.e., OxPhos or anaerobic glycolysis, is closely related to proliferation and osteogenesis [32], the bioenergetic assay may shed more light on metabolic regulation during succinate supplementation in hPDLCs as well as other MSCs. Moreover, interaction of HIF-1*α* with the Notch and nuclear factor-*κ*B pathway during osteogenesis needs to be further studied during succinate supplementation.

Extracellular succinate may bind to its specific receptor GPR91, transmitting metabolic signal outside into cytosol and nucleus [41]. GPR91 activation by succinate leads to an influx of calcium and signaling via mitogen-activated protein kinase (MAPK) and protein kinase C (*PKC*) pathway, leading to an increase in secretion of NO and production of ROS [34], which may promote inflammatory response and enhance cell proliferation [42]. In our present study, we observed elevated levels of succinate in hPDLCs both in the cytosol and culture supernatant, which upregulate GPR91 expression. Knockdown of GPR91 reduced hypoxia-mediated proliferation, suggesting that hypoxia-induced proliferation was dependent on GPR91.

Succinate moves freely between the mitochondria and the cytosol via the dicarboxylic acid translocator in the mitochondrial inner membrane and the voltage-dependent anion channel (VDAC/porin) in the mitochondrial outer membrane [19]. It has been shown that extracellular succinate can be transported into cytosol and mitochondria by the plasma membrane-bound Na^+^-dependent dicarboxylic acid transporter NaDC3 (SLC13A3 gene) [43]. Therefore, the supplement of succinate (5 mM) may promote NaDC3-mediated intracellular transportation, leading to constant succinate accumulation in the cytosol and mitochondria, which clearly explained how the succinate supplement promotes cell proliferation of PDLFs in the present study. Such transmembrane movement by NaDC3 indicated that the principal mechanism for succinate supplement-enhanced proliferation is its entrance into the cytosol to stabilize HIF-1*α*, which is different from GPR91-dependent pathway in the hypoxia condition.

The supplement of succinate is similar to succinate metabolism in the ischemia-reperfusion model, and elevated succinate may upregulate SDH transcription by substrate-enzyme interaction [44], leading to enhanced SDH transcription as Figure 4(d). As shown in Figure 4(a), intracellular succinate levels peaked at 6 h; such high concentration of intracellular succinate may significantly affect its protein expression and activity by several possible mechanisms: succinate may stabilize HIF-1*α* to mimic hypoxic state, which triggers hypoxia-inducible C-to-U coding RNA editing [45], and miRNA-mediated epigenetic regulation may also contribute to transcription modification [46]; in addition, extensive ROS generation by reverse electron transport (RET) at mitochondrial complex I during oxidation of succinate may critically affect the availability of reduced NAD^+^ and altered activity of SIRT3, a NAD-dependent deacetylase that affects SDH enzymatic activity [47]; moreover, succinate accumulation in the mitochondrial may increase levels of itaconate, which further inhibit SDH activity [46].

In conclusion, we observed that hypoxia promotes hPDLC proliferation, migration, and osteogenic differentiation through intracellular succinate-induced HIF-1*α* stabilization and extracellular succinate-caused GPR91 activation. Moreover, exogenous succinate can act as a mimic signal, causing a “pseudohypoxia” condition to enhance proliferation of hPDLCs. Our present study discovered that succinate, a metabolite of the TCA cycle, can be utilized to fuel the glycolysis in the condition of normal oxygen.

## Figures and Tables

**Table 1:** Nucleotide sequence of primers used in real-time PCR.

Gene	Forward primer (5′ to 3′)	Reverse primer (5′ to 3′)
CCND1	CAATGACCCCGCACGATTTC	CATGGAGGGCGGATTGGAA
CCNB1	AACTTTCGCCTGAGCCTATTTT	TTGGTCTGACTGCTTGCTCTT
CCND3	TACCCGCCATCCATGATCG	AGGCAGTCCACTTCAGTGC
P21	TGTCCGTCAGAACCCATGC	AAAGTCGAAGTTCCATCGCTC
OCN	GGCAGCGAGGTAGTGAAGAG	GATGTGGTCAGCCAACTCGT
ALP	GACCCTTGACCCCCACAAT	GCTCGTACTGCATGTCCCCT
RUNX2	GGAGTGGACGAGGCAAGAGTTT	AGCTTCTGTCTGTGCCTTCTGG
COL-1	AGAACAGCGTGGCCT	TCCGGTGTGACTCGT
HK2	GAGCCACCACTCACCCTACT	CCAGGCATTCGGCAATGTG
PFKFB3	TTGGCGTCCCCACAAAAGT	AGTTGTAGGAGCTGTACTGCTT
SDHa	CAGCATGTGTTACCAAGCTGT	GGTGTCGTAGAAATGCCACCT
SDHb	ACCTTCCGAAGATCATGCAGA	GTGCAAGCTAGAGTGTTGCCT
GPR91	GGAGACCCCAACTACAACCTC	AGCAACCTGCCTATTCCTCTG
*β*-Actin	GTGGGGCGCCCCAGGCACCA	CGGTTGGCCTTGGGGTTCAGGGGGG

## Data Availability

The data used to support the findings of this study are available from the corresponding author upon request.
